# Barriers to biomedical care: Qualitative insights into health-seeking behaviors and systemic challenges among people affected by leprosy in Tanzanian communities

**DOI:** 10.1371/journal.pntd.0014806

**Published:** 2026-10-15

**Authors:** Sylivester Asimwe Muhigi, Kijakazi Mashoto, Mercy Chiduo

**Affiliations:** 1 National Institute for Medical Research, Muhimbili Research Centre, Dar es Salaam, Tanzania; 2 National Institute for Medical Research, Headquarters, Dodoma, Tanzania; 3 National Institute for Medical Research, Dodoma Medical Research Centre, Dodoma, Tanzania; Marie Adelaide Leprosy Center, PAKISTAN

## Abstract

**Background:**

Delayed diagnosis in leprosy reflects both how people interpret symptoms and seek care, and how effectively health systems recognize and manage the disease. Qualitative evidence from Tanzania remains limited on how the interaction between patients and health care workers affect provision of care to people affected by leprosy in a timely manner. The main aim of this study was to explore health-seeking behaviors and systemic challenges affecting access to biomedical care among people affected by leprosy in selected Tanzanian communities.

**Methods:**

Ethics Statement: Ethical approval for the study was obtained from the Medical Research Coordinating Committee (MRCC) through the National Health Research Ethics Committee (NatHREC) with reference number NIMR/HQ/R.8a/Vol.IX/4587. Written informed consent was obtained from all participants before data collection. Identifying information was removed from transcripts, data were anonymized before analysis, and audio files and transcripts were stored on password-protected computers accessible only to the data manager and the principal investigator. Data will be retained for up to 10 years per NIMR policy. An anonymized coding framework and de-identified extracts are available upon reasonable request. Given the sensitive nature of gender-based violence disclosures, referral procedures were established to ensure that participants requiring additional support could be linked to appropriate services This qualitative study was conducted in Tanga and Morogoro regions, Tanzania. Semi-structured interviews and focus group discussions were held with people affected by leprosy, community members, District Tuberculosis and Leprosy Coordinators, Community Health Workers, and Prevention of Disability officers. Data were audio-recorded, transcribed, translated into English, and analyzed thematically.

**Results:**

Our findings revealed various factors which contributed to delays in seeking for biomedical care among people affected with leprosy. First, early symptoms were often normalized or attributed to witchcraft and curses, prompting initial care from traditional healers. Second, stigma, fear of rejection, and advice from relatives and community members delayed disclosure and transition to biomedical care. Third, health-system barriers including misdiagnosis, limited provider familiarity with leprosy, referral delays, medicine shortages, and inadequate support for disability management delayed confirmation of diagnosis and initiation of appropriate treatment.

**Conclusion:**

Health-seeking behaviors among people affected by leprosy were shaped by stigma, social influence, and health-system barriers that delayed or limited access to biomedical care. Evidence-based interventions, including culturally sensitive education, stigma reduction, and strengthening of frontline biomedical services, can help overcome these barriers. Policy efforts should prioritize biomedical care alongside community-based awareness to reduce stigma and promote timely care-seeking.

## Author summary

Leprosy is a curable infectious disease, yet many people affected by it in Tanzania present to health facilities late, often after the disease has already caused disability. We interviewed 56 people, including people affected by leprosy, community members, community health workers, and leprosy programme coordinators in the Tanga and Morogoro regions, to understand why delays in seeking biomedical care occur. We found three connected barriers. First, early symptoms were often interpreted as ordinary skin problems or as the result of witchcraft or curses, leading many people to seek care first from traditional healers. Second, fear of stigma, rejection by spouses, and advice from relatives delayed disclosure and the move to biomedical care. Third, even after reaching a health facility, misdiagnosis (often as a fungal infection), drug shortages, and weak referral systems delayed correct treatment. These findings show that reducing leprosy-related disability requires not only medical services but also community education to change beliefs and reduce stigma, and strengthening of frontline health services to correctly diagnose leprosy early and reliably provide drugs.

## Introduction

Leprosy, or Hansen’s disease, is a chronic infectious disease caused mainly by Mycobacterium leprae that affects the skin, peripheral nerves, upper respiratory mucosa, and eyes. The disease is curable, however, if left untreated, it can lead to progressive and permanent disability, which can often be prevented through early diagnosis and multidrug therapy [1]. The World Health Organization (WHO) reported 172,717 new leprosy cases globally in 2024, including 19,171 in the African Region; Tanzania remains one of six African countries reporting more than 1,000 new cases annually [2]. It is reported that, in Tanzania, male above 15 years affected by leprosy and living with HIV, with previous leprosy treatment history, have been identified as independent risk factors for leprosy-related disability, further highlighting the consequences of late detection [3]. Baseline evidence from Tanzanian Post-Exposure Prophylaxis for Leprosy (PEP4LEP) districts also documented long delays to diagnosis, with a mean case-detection delay of 28.1 months and a median delay of 21.5 months [4].

Health-seeking behavior is central to how people interpret symptoms, decide whether to disclose illness, choose sources of care, and ultimately engage with biomedical services [5,6]. In leprosy-endemic settings, the pathway to treatment is often non-linear and may include self-medication, traditional or religious healing, and long delays before presentation to a health facility [7–9]. Studies from Ethiopia and other endemic settings have documented prolonged case-detection delays, frequent initial consultation with traditional healers, and worsening disability among those who present late [10,11].

Delayed care-seeking in leprosy is influenced by multiple factors beyond individual choice. At the patient and community level, explanatory beliefs, anticipated stigma, household and community influence, and competing social obligations may delay recognition of symptoms and presentation to biomedical care [12,13]. A systematic review on gender and leprosy-related stigma found that women often experience greater concealment, social rejection, and treatment delay, underscoring how social position can influence access to care [14]. At the health-system level, limited leprosy-specific expertise among frontline and referral health care workers, referral delays, medicine shortages, and costs related to transport or prescribed medicines may further delay diagnosis, interrupt treatment, and limit access to disability-related care [13].

Current regional and global initiatives emphasize integrated skin screening, early case detection, prevention of disability, stigma reduction, and stronger frontline diagnostic capacity, especially in resource-constrained settings [15,16]. However, there remains limited evidence from Tanzania on how people affected by leprosy interpret symptoms, navigate care, and experience the formal health system. This makes studying the interaction between health-seeking behavior and systemic barriers among people affected by leprosy crucial. In this study, biomedical care refers to formal health services provided by trained medical professionals within the formal health system, including clinical assessment, diagnosis, multidrug therapy, referral, and disability-related care, distinguished from traditional, religious, or informal care. This study therefore explored health-seeking behaviors and systemic challenges affecting access to biomedical care among people affected by leprosy in selected Tanzanian communities. Specifically, this study was guided by the following research questions:

1. How do individual and cultural factors shape the initial interpretation of leprosy symptoms and the decision to seek biomedical care?
2. How do social influences and stigma affect disclosure and the transition to formal health services?
3. What health-system barriers delay accurate diagnosis and treatment once patients enter biomedical care?

## Methodology

### Study design

We present findings from a qualitative descriptive study conducted in the Tanga and Morogoro regions of Tanzania. We conducted in-depth interviews (IDIs), key informant interviews (KIIs), and focus group discussions (FGDs) with people affected by leprosy (PALs), community members, District Tuberculosis and Leprosy Coordinators (DTLCs), Community Health Workers (CHWs), and Prevention of Disability (PoD) officers. To ensure privacy and minimize power dynamics, all sessions were conducted in private settings, and facility leprosy coordinators or other health staff were not present during the interviews with PALs or community members..

### Analytical framework

To frame data collection and analysis, we adapted the three-delays model to the leprosy care pathway. This framework allowed us to systematically categorize barriers across the entire patient journey, highlighting that delays are not solely patient-driven but are compounded by systemic failures (Table 1).

**Table 1:** Adapted three-delays model framework for leprosy care.

Delay Phase	Definition in Leprosy Context	Corresponding Study Theme
**Delay 1**	Period from symptom onset to the decision to seek biomedical care, shaped by symptom interpretation, stigma, cultural beliefs, and preference for traditional care.	Theme 1: Preference for traditional care as the first response to leprosy symptoms.
**Delay 2**	Barriers to reaching biomedical services, including distance, cost, referral difficulties, and limited disclosure due to fear of stigma.	Theme 2: Stigma, fear of rejection, and social influence delaying disclosure and transition to biomedical care.
**Delay 3**	Barriers to receiving adequate care once within the formal health system, including misdiagnosis, limited provider familiarity with leprosy, medicine shortages, and inadequate disability services.	Theme 3: Health-system barriers delaying diagnosis and treatment after entry into biomedical care.

### Study setting

This study was conducted in two regions of Tanzania; Tanga and Morogoro. In each region, rural and urban districts (Tanga and Muheza) in Tanga, and (Morogoro and Mvomero) in Morogoro, known to be endemic areas for leprosy were selected (Fig 1). These regions have consistently reported higher prevalence rates of the disease compared to other parts of the country, and Morogoro and Mvomero district councils consistently notify among the highest number of leprosy cases nationally [3]. Furthermore, these districts are part of the Tanzanian Post-Exposure Prophylaxis for Leprosy (PEP4LEP) implementation area, where baseline studies have documented prolonged case-detection delays [4]. They were therefore selected as critical sites for understanding the impacts of leprosy and the barriers to timely care among the affected populations.

**Fig 1:**
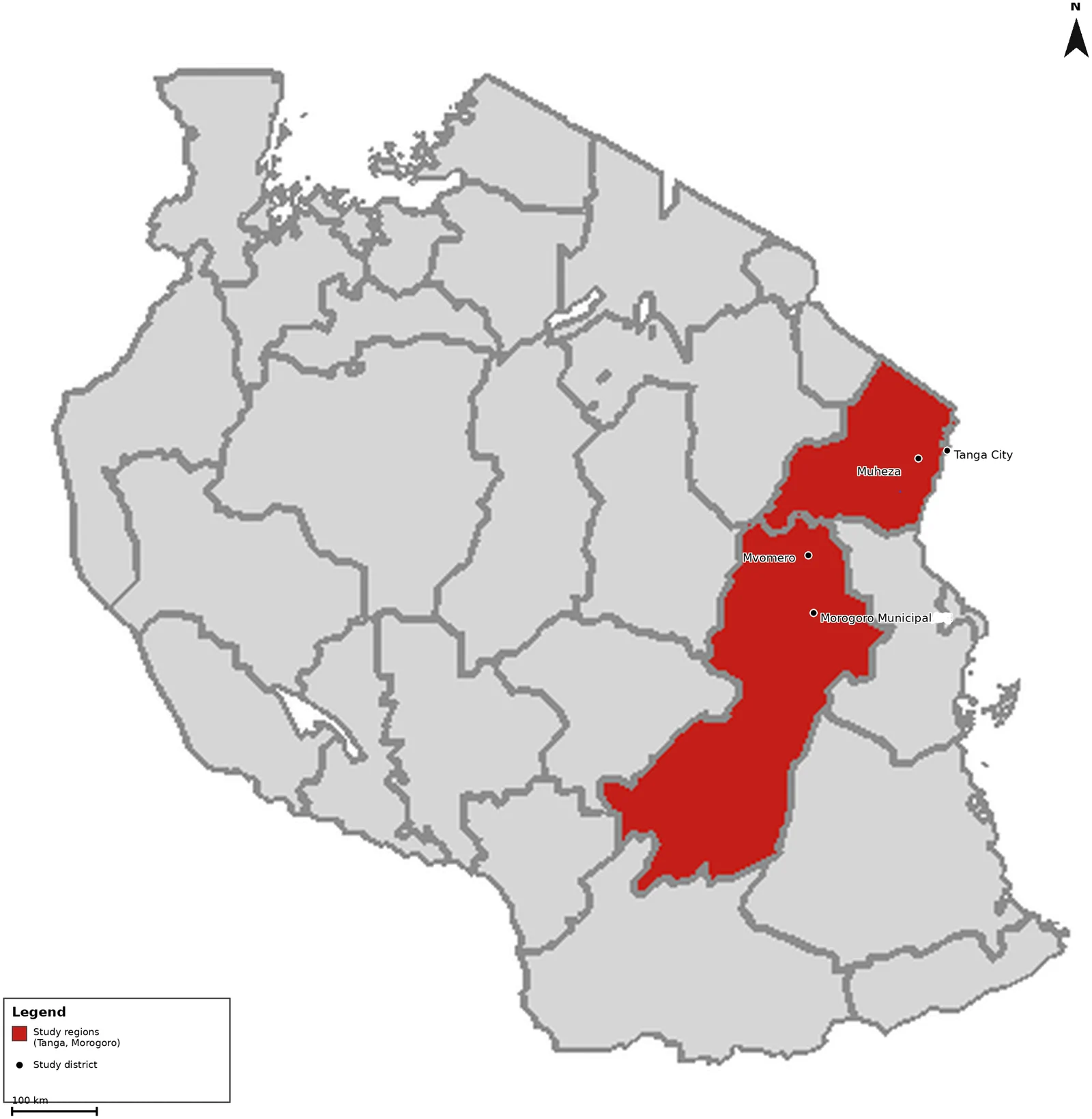
Map of the study area showing the Tanga and Morogoro regions and the four study districts. Base map: simplemaps.com, https://simplemaps.com/svg/country/tz, license terms: https://simplemaps.com/resources/svg-license: underlying GIS boundary data available under CC BY 4.0: https://simplemaps.com/resources/gis.

### Study participants and sampling

The study population included people affected by leprosy, community members not affected by leprosy, District Tuberculosis and Leprosy Coordinators (DTLCs), Community Health Workers (CHWs), and Prevention of Disability (PoD) officers. The inclusion criteria were people affected by leprosy aged 18 years or older who were receiving or had completed multidrug therapy, community members residing in proximity to persons affected by leprosy, CHWs and PoD officers providing services to this population, and DTLCs from the study districts (Table 4). Consistent with ethical approval, only individuals able and willing to provide written informed consent were enrolled. Purposive sampling was used to capture maximum variation across sex, age, urban and rural residence, education level, and participant role.

With the assistance of facility leprosy coordinators, people affected by leprosy were identified from facility leprosy registries and the broader mixed-methods study database, and those who reported gender-based violence experience in the quantitative survey were purposively selected and invited for in-depth interviews. Community members were recruited with the assistance of village and street leaders in collaboration with CHWs and PoD officers. DTLCs were selected based on their coordination roles. The composition of the sample, comprising 20 in-depth interviews with people affected by leprosy, 4 key informant interviews with DTLCs, and 4 focus group discussions (two with community members and two with CHWs and PoD officers), was designed to triangulate lived experience, community narratives, and service-delivery perspectives. Sociodemographic and economic details such as education, income, and religion, as well as disease duration, were not systematically collected for the qualitative participants; the socioeconomic profile of the broader quantitative cohort is cited as context.

### Data collection

Data were collected between July and August 2024 through semi-structured key informant interviews (KIIs) and focus group discussions (FGDs). Before data collection, research assistants and experienced anthropologists participated in the study protocol training. The interview and discussion guides (S1 and S2 Appendices) were adapted from the WHO Researching Violence Against Women toolkit and the guidelines on Social Problems of Women Leprosy Patients, developed for the parent mixed-methods study, and piloted in Tanga City before use. Potential participants identified from facility records were contacted in advance by facility leprosy coordinators and invited to participate at the coordinator’s office or a designated health facility.

A total of 24 individual interviews (20 IDIs with people affected by leprosy and 4 KIIs with DTLCs) and 4 FGDs of 8 participants each (two with community members and two with CHWs and PoD officers, one per district) were conducted to capture lived experience, community narratives, and service-level perspectives.

Interviews and discussions were conducted using guides focused on knowledge and perceptions of leprosy, symptom recognition, care-seeking pathways, stigma and disclosure, cultural and gender norms, experiences with healthcare workers, referral processes, logistical barriers, and structural challenges affecting access to biomedical care. Sessions lasted approximately 30–50 minutes, were audio-recorded upon consent, and were conducted in private settings to protect confidentiality and participant comfort.

### Researcher positionality and reflexivity

The interviewers were independent researchers from the National Institute for Medical Research (NIMR) and were not known to participants. No team member had prior involvement in leprosy control or research in the study communities, and leprosy coordinators and facility staff did not sit in on interviews. Interviews were conducted in private settings using semi-structured guides with neutral, non-leading probing. As public health researchers, we acknowledge that our positions may have shaped probing and interpretation; analysis was therefore repeatedly checked against participants’ own accounts

### Data management and analysis

Interviews were conducted in Kiswahili by bilingual interviewers. Audio recordings were transcribed verbatim in Kiswahili and subsequently translated into English by a trained interpreter fluent in both languages. Translated transcripts were reviewed with the primary researcher to clarify culturally embedded terms and concepts. All transcripts were anonymised before analysis to protect participant confidentiality.

Two researchers coded the data, and disagreements were resolved through discussion and consensus. A formal codebook was not developed a priori; the coding framework was generated inductively and refined iteratively. Data were managed in NVivo version 14. Analysis was inductive and thematic: transcripts were read repeatedly, initial open codes were generated, similar codes were grouped into thematic categories, and supporting quotations were extracted; themes and sub-themes were organised in matrices according to the study sub-questions. Codes were compared across participant groups, study sites, sex, and urban/rural residence, and emerging codes captured issues such as stigma, gendered experiences of disclosure and care-seeking, cultural misconceptions, referral delays, resource shortages, and limited health-worker familiarity with leprosy. The final thematic structure was reviewed against the study objectives. Formal member checking and negative-case analysis were not conducted; this is acknowledged as a limitation.

## Results

A total of 56 participants took part: 24 individual interviews (20 IDIs with people affected by leprosy and 4 KIIs with DTLCs) and 4 FGDs of 8 participants each, conducted across the four study districts (Tables 1–4). Three interrelated themes captured the main barriers to biomedical care: (1) preference for traditional care as the first response to leprosy symptoms; (2) stigma, fear of rejection, and social influence delayed disclosure and transition to biomedical care and (3) health-system barriers delayed diagnosis and treatment after entry into biomedical care. These themes map onto the adapted three-delays model: Theme 1 corresponds to Delay 1 (symptom onset to the decision to seek biomedical care), Theme 2 to Delay 2 (reaching biomedical services), and Theme 3 to Delay 3 (receiving adequate care) (Fig 2).

**Table 2:** Characteristics of qualitative participants.

Variable	PALs (n = 20)	DTLCs (n = 4)	Community members (n = 16)	CHWs/PoDs (n = 16)	Total (n = 56)
**Sex**					
Female	9 (45.0)	3 (75.0)	8 (50.0)	8 (50.0)	28 (50.0)
Male	11 (55.0)	1 (25.0)	8 (50.0)	8 (50.0)	28 (50.0)
**Age range (years)**	22–88	30–59	27–66	23–55	22–88
**Marital status**					
Married	8 (40.0)	3 (75.0)	7 (43.8)	9 (56.3)	27 (48.2)
Single	3 (15.0)	1 (25.0)	3 (18.8)	5 (31.3)	12 (21.4)
Separated	2 (10.0)	0 (0.0)	4 (25.0)	1 (6.3)	7 (12.5)
Divorced	2 (10.0)	0 (0.0)	0 (0.0)	0 (0.0)	2 (3.6)
Widowed	2 (10.0)	0 (0.0)	2 (12.5)	0 (0.0)	4 (7.1)
Cohabiting	2 (10.0)	0 (0.0)	0 (0.0)	0 (0.0)	2 (3.6)
**Occupation**					
Farmer	9 (45.0)	0 (0.0)	5 (31.3)	3 (18.8)	17 (30.4)
Self-employed	8 (40.0)	0 (0.0)	8 (50.0)	10 (62.5)	26 (46.4)
Formal employment	0 (0.0)	4 (100.0)	2 (12.5)	0 (0.0)	6 (10.7)
Driver	0 (0.0)	0 (0.0)	0 (0.0)	2 (12.5)	2 (3.6)
Business	0 (0.0)	0 (0.0)	0 (0.0)	1 (6.3)	1 (1.8)
Beggar	1 (5.0)	0 (0.0)	0 (0.0)	0 (0.0)	1 (1.8)

Data are n (%). PAL, person affected by leprosy; DTLC, District Tuberculosis and Leprosy Coordinator; CHW, Community Health Worker; PoD, Prevention of Disability officer. Marital status was not reported for two participants and occupation for three

**Table 3:** Qualitative data collection by district.

District	Quantitative			Qualitative			
	Male	Female	Total	IDIs – PALs	KIIs – DTLCs	FGD (8 participants)	Qualitative – Total
Tanga City (urban)	18	4	22	5	1	1	14
Muheza (rural)	20	14	34	5	1	1	14
Morogoro Municipal/DC (urban)	55	37	92	5	1	1	14
Mvomero (rural)	56	50	106	5	1	1	14
**Total**	**149**	**105**	**254**	**20**	**4**	**4 (n = 32)**	**56**

*One FGD (8 participants) was conducted per district; across districts, two FGDs were held with community members (n = 16) and two with CHWs/PoD officers (n = 16). All IDIs, KIIs, and FGDs lasted 30–50 minutes.*

**Table 4:** Study population and inclusion criteria.

Participant group	Inclusion criteria	Data collection method
**People affected by leprosy**	Aged ≥18 years; affected by leprosy; receiving or having completed multidrug therapy; experienced GBV (identified from the quantitative study database); resident in study districts	IDI
**District TB and Leprosy Coordinators**	Responsible for leprosy coordination in the study districts	KII
**Community members**	Resident in study communities near persons affected by leprosy; not necessarily affected by leprosy	FGD
**Community Health Workers / Prevention of Disability officers**	Providing community health or disability-related services to people affected by leprosy in the study districts	FGD

*Consistent with ethical approval, only individuals able and willing to provide written informed consent were enrolled.*

**Fig 2:**
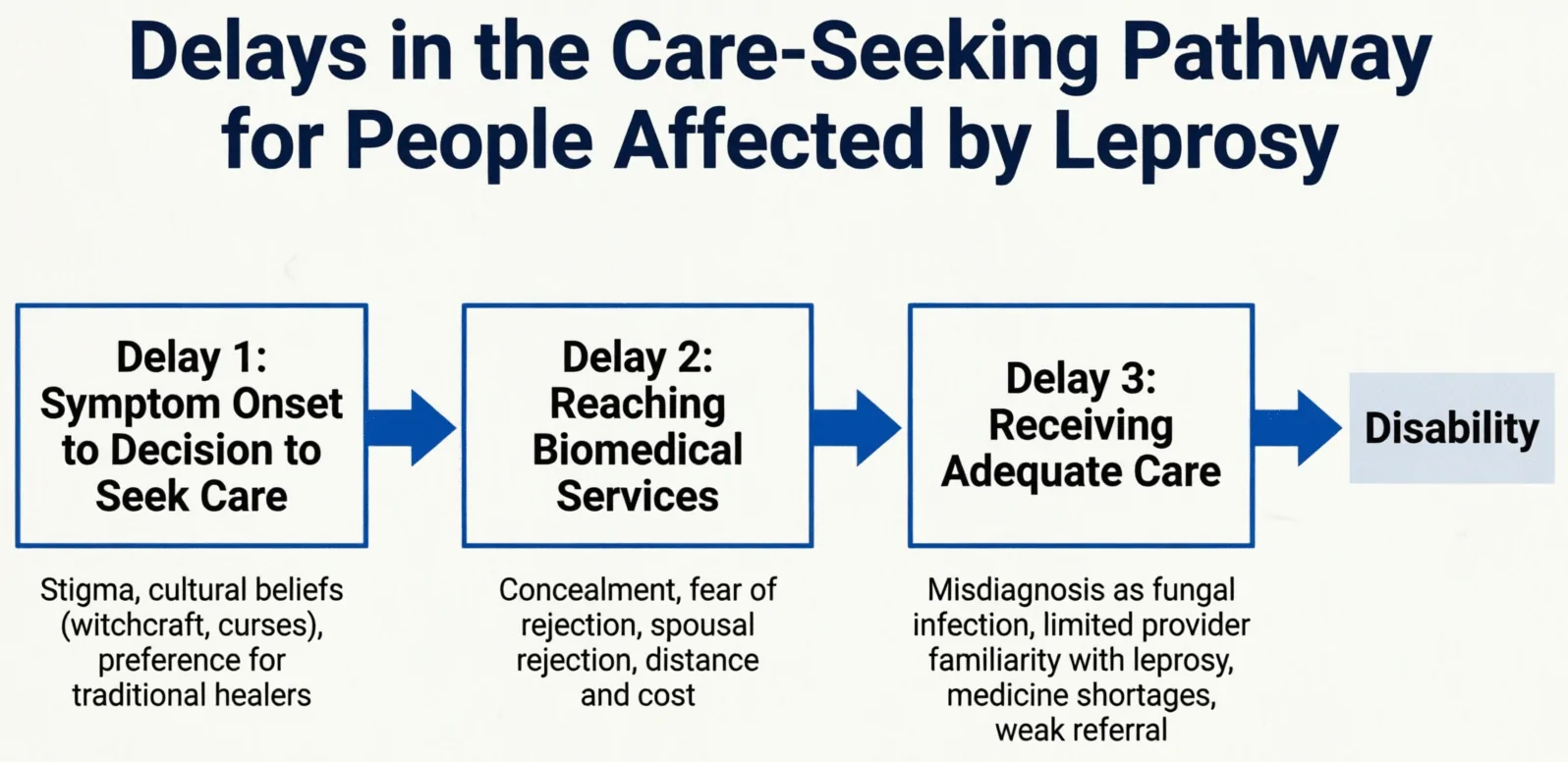
Conceptual framework of barriers to biomedical care mapped onto the adapted three-delays model.

These three themes do not operate in isolation; rather, they represent a cascading pathway of compounding delays. Initial reliance on traditional care due to symptom misinterpretation (Theme 1) is frequently reinforced by community stigma and fear of disclosure (Theme 2). This social pressure delays help-seeking, ultimately leading to late presentation at health facilities. By the time patients enter the formal system, their advanced symptoms encounter systemic weaknesses, such as misdiagnosis and resource shortages, which further compound the delay in receiving adequate care (Theme 3).

1. Preference for traditional care as the first response to leprosy symptoms

Many participants did not initially interpret early signs of leprosy as a disease requiring biomedical care. Skin patches, numbness, swelling, itching, or weakness were frequently normalized, mistaken for ordinary skin conditions, or explained through local belief systems such as witchcraft, curses, heredity, or spiritual attack. These interpretations shaped the first response to illness and often made traditional healers appear more appropriate than health facilities.

*"The first step I took... I went to traditional healers. I went to three traditional healers."* (female participant, affected by leprosy, IDI, Morogoro District Council n=20)

*"They kept telling me that I was bewitched. So, I was sent to seven different healers. Seven chickens were spent."* (female participant, affected by leprosy, IDI, Morogoro rural n=20)

Such narratives show that the preference for traditional care shaped not only the first point of consultation but also the timing of biomedical presentation. In this context, delayed care often results from a preference for traditional healing that persists until individuals recognize their symptoms as requiring biomedical attention.

2. Stigma, fear of rejection, and social influence delayed disclosure and transition to biomedical.

Decisions were strongly influenced by family members, neighbors, and wider community narratives. Advice to seek traditional care often persisted even when the diseases worsened. Fear of being labelled, rejected, or humiliated also constrained disclosure and delayed movement into care. For some women in particular, the possibility of spousal rejection made disclosure especially difficult.

*"Being treated by traditional healers is what delayed me. I was not supposed to get this disability."* (female participant, affected by leprosy, IDI, Morogoro rural n=20)

*"After failing to get better from the traditional healer, that is when I came to the hospital. Even now, people keep advising me to go back to a traditional healer, but I always tell them this disease cannot be treated by traditional healers."* (male participant, affected by leprosy, IDI, Tanga urban n=20)

### Gendered experiences of stigma and care-seeking

Spousal rejection emerged as an important gendered manifestation of leprosy-related stigma that influenced disclosure and care-seeking. Female participants described refusal of intimacy, accusations of infecting spouses, abandonment, and pressure to separate, which made disclosure difficult and, in some cases, delayed movement into biomedical care.

"We were not having intimacy until I took him to the hospital where he was given information on leprosy; that is when he changed." (female person affected by leprosy, IDI, Morogoro Municipal n=20)

"My husband died thinking I was the one who infected him with leprosy. He even ran away and married another woman." (female person affected by leprosy, IDI, Morogoro Municipal n=20)

In contrast, male participants more frequently described external community pressure and expectations to consult traditional healers before considering biomedical options, rather than intimate partner rejection.

These suggest that misdiagnosis was not only a matter of lack of knowledge, but also the result of repeated movement across different therapeutic options before biomedical care was accepted as credible and necessary.

3. Health-system barriers delayed diagnosis and treatment after entry into biomedical care

Reaching a health facility did not necessarily lead to prompt diagnosis or effective care. Participants described repeated consultations, misdiagnosis as fungal infection or other skin disease, and prolonged treatment journeys before leprosy was recognized. Health care workers also acknowledged that low familiarity with leprosy at primary-care level contributed to misdiagnosis.

*"The hospitals I visited first, some told me it was a fungal skin disease. They injected me and prescribed some medicines, but the problem did not go away."* (female participant, affected by leprosy, IDI, Morogoro Municipal n=20)

*"If a health worker sees the patient, he or she may think it is a normal skin condition, perhaps a fungal infection, and treat for that first."* (district Tuberculosis and leprosy coordinator, KII, Morogoro n=4)

Participants and health care workers also described referral bottlenecks, lack of diagnostic tools for confirmation, medicine shortages, and lack of specialist services for disability management. For some participants, distance to facilities and the cost of repeated visits compounded these delays. Others reported fear of humiliating language or blame within healthcare facilities, which further discouraged timely engagement with services.

*"The problem in treating these patients is mainly medicines. A patient is given prednisolone for a long time, but if they cannot afford it, they take it intermittently and do not get results."* (district Tuberculosis and coordinator, KII, Muheza n=4)

*"Leprosy patients are afraid of seeking medical care because they will be humiliated and spoken to with hurtful words."* (female participant, affected by leprosy, IDI, Morogoro n=20)

Despite these barriers, several participants described hospital leprosy treatment as effective once the proper diagnosis was made and treatment was started.

### Variations in perspectives across participant groups

Perspectives differed by participant group. People affected by leprosy emphasized concealment of symptoms, fear of stigma, and initial reliance on traditional healers. Community members and community leaders emphasized cultural explanations, including witchcraft, curses, and contagion, and described fear of close contact with affected persons. Health workers and coordinators focused on diagnostic challenges, particularly misdiagnosis as fungal infection, medicine shortages, and referral and laboratory constraints. Female participants more commonly described marital tension and spousal rejection, whereas male participants described community pressure to consult traditional healers.

## Discussion

This study shows that barriers to biomedical care among people affected by leprosy in selected Tanzanian communities are related to interaction of preference for traditional care, social influence, stigma, and health-system barriers. Many participants initially interpreted early signs of leprosy through local explanatory beliefs, including witchcraft, curses, heredity, spiritual attack, or common skin conditions. These interpretations made traditional care appear more appropriate than biomedical treatment and contributed to repeated movement through informal care before participants reached health facilities. This pattern is consistent with other studies elsewhere showing that traditional care, delayed presentation, and long diagnostic delays remain common in leprosy-endemic settings [10,11]. The findings also resonate with baseline data from Tanzania (Morogoro, Mvomero, and Lindi Districts) where prolonged case-detection delay was documented [4].

While previous studies have documented traditional care-seeking and stigma in leprosy-endemic settings [10,11,14], this study adds qualitative evidence from Tanzanian communities collected after the PEP4LEP baseline assessment, providing insight into persistent barriers in the post-baseline implementation phase [4,15]. By triangulating the perspectives of people affected by leprosy, community members, and health-system actors, the findings demonstrate that delays are not produced by patient beliefs alone; rather, community stigma and health-system weaknesses, particularly the frequent misdiagnosis of leprosy as a fungal infection, interact to prolong the pathway to accurate diagnosis and appropriate treatment.

Our results also highlight stigma as a practical barrier to care rather than only a social consequence of disease. Stigma operated at multiple levels, including within families, communities, and healthcare encounters. Fear of rejection, concealment of symptoms, concern about being seen at a facility, and fear of humiliation shaped when and how participants sought help. For some participants, advice from relatives and community members sustained reliance on traditional healing even after symptoms worsened, while fear of spousal rejection made disclosure particularly difficult for some women. This is consistent with evidence that stigma contributes to treatment delay and that women may be especially vulnerable to concealment, social exclusion, and delayed help-seeking in leprosy-affected settings [11,14].

The findings also show that treatment delays continued even upon visiting the health facilities. Participants described repeated consultations, misdiagnosis as fungal infection or other skin disease, weak diagnostic capacity at primary-care level, and referral processes that depended on scarce leprosy-specific expertise. These findings show that delayed and or misdiagnosis linked with late health seeking behavior as well as limitations in the health system’s ability for early leprosy diagnosis and management. They suggest that the central challenge is not the effectiveness of biomedical care itself, but rather the delayed recognition, frequent misdiagnosis, and delayed initiation of accurate treatment once patients enter the formal health system. Medicine shortages and stockouts at health facilities, inability to afford prescribed medicines such as prednisolone among people affected with leprosy, travel costs, and limited specialist services for disability management further constrained adherence to care. These findings align with broader evidence that dermatology and skin-NTD capacity remain limited in many resource-constrained settings and that health care workers often lack the support needed to early recognize uncommon or stigmatized skin conditions [16].

These findings suggest that early diagnosis will require action at community, district, regional, and national levels. At the community level, interventions should address misconceptions about causes of leprosy and its transmission, reduce stigma, including spousal rejection and fear of transmission within marriage, and promote recognition of early signs and symptoms such as numb patches, sensory loss, and non-healing lesions, with traditional healers engaged and sensitized on early leprosy signs and referral pathways, respecting community care-seeking practices, rather than viewed solely as barriers.

At the district level, frontline workers should be trained with simple job aids to distinguish leprosy from fungal and other skin conditions, referral links between dispensaries, health centres, and district hospitals should be strengthened with functional referral SOPs, and Community Health Workers and local leaders should be engaged in early symptom recognition. At the regional level, referral hospitals and district teams in high-burden regions such as Tanga and Morogoro need continued mentoring in integrated skin screening and stronger links to disability-related care.

At the national level, leprosy and skin-NTD screening should be built into primary care training, supplies of multidrug therapy and prednisolone secured, and guidance issued on engaging traditional healers in early case detection and referral. Reducing direct costs associated with repeated facility visits, transport, and prescribed medicines may also support continuity of care after diagnosis. To reach people who remain undiagnosed, socially isolated, or unregistered, programmes should use community-based active case-finding, household contact screening, mobile outreach, and integration with skin-NTD initiatives, and collaboration with local leaders, CHWs, and traditional healers. Future work should quantify patient, health-system, and diagnostic delays, test healer-engagement and gender-sensitive stigma-reduction models, and examine implementation of medicine supply and referral completion. These recommendations are consistent with the preventive and case-finding approach underpinning Post-Exposure Prophylaxis for Leprosy (PEP4LEP) and related zero-leprosy strategies [15,17].

## Strengths and limitations

A major strength of this study is its focus on qualitative evidence relevant to health-seeking behavior and systemic barriers, which improves coherence. A key strength of applying the adapted three-delays model is that it moves beyond framing delays as solely the result of individual patient choices or beliefs. Instead, it provides a structured framework to systematically categorize barriers across the entire patient journey, highlighting how community-level stigma and health-system failures interact with individual decision-making to produce compounding delays. This approach allowed us to identify actionable intervention points at multiple levels (community, facility, and system) rather than attributing delayed diagnosis exclusively to patient-level factors.

The inclusion of multiple participant groups, namely people affected by leprosy, community members, CHWs, PoD officers, and DTLCs, allowed triangulation across lived experience, community narratives, and service-delivery perspectives. A further strength is the transdisciplinary nature of the study, which brought together public health, clinical, sociological, and community-based perspectives.

This study also has limitations. As a purposively sampled qualitative study conducted in four districts, the findings are not statistically generalizable to all people affected by leprosy in Tanzania. In addition, because this manuscript draws only on the qualitative component of the broader study, it cannot estimate the prevalence of specific barriers. Because discussions addressed stigma, witchcraft beliefs, and experiences of humiliation at facilities, responses may have been subject to social desirability bias.

Recruitment through existing leprosy coordination structures may under-represent people affected by leprosy who are undiagnosed, socially isolated, not registered, or outside formal care networks. Formal member checking and negative-case analysis were not conducted. Disease duration, religion, and disability status were not collected for qualitative participants; education and socioeconomic characteristics are available in the broader quantitative study and were cited as context. Data collection was not concluded on the basis of a formal thematic saturation criterion; sample adequacy was instead guided by information power.

## Conclusion

Health-seeking behavior and systemic barriers are deeply intertwined in the pathway to leprosy diagnosis and treatment. Among people affected by leprosy in these Tanzanian communities, delayed biomedical care was shaped by preference for traditional care, fear of stigma, weak disclosure, and shortcomings within the formal health system. Reducing disability and delayed diagnosis will therefore require a combined response: stronger community education and stigma reduction, earlier integrated case-finding, better prepared health workers, reliable medicines, and more functional referral and disability-care pathways.

## Supporting information

S1 Appendix In-depth interview guide for people affected by leprosy. (DOCX)

S2 Appendix Key informant interview guide for District Tuberculosis and Leprosy Coordinators, and focus group discussion guide for Community Health Workers and community members. (DOCX)

## References

[1] World Health Organization (WHO). Leprosy [Internet]. Geneva: WHO; 2024 [cited 2026 May 7]. Available from: https://www.who.int/news-room/fact-sheets/detail/leprosy

[2] WHO. Leprosy. WHO. https://www.afro.who.int/health-topics/leprosy 2024. Accessed 2026 May 7.

[3] Mrema G, Hussein A, Magoge W, Mmbaga V, Simba A, Balama R, et al. Burden of leprosy and associated risk factors for disabilities in Tanzania from 2017 to 2020. PLoS One. 2024;19(10):e0311676. doi: 10.1371/journal.pone.0311676 39388437 PMC11466380

[4] Mwageni N, Kamara D, Kisonga R, Njako B, Nyakato P, Pegwa A. Leprosy epidemiological trends and diagnosis delay in three districts of Tanzania: A baseline study. Lepr Rev. 2022;93(3):209–23. doi: 10.47276/lr.93.3.209

[5] Singh S, Sinha AK, Banerjee BG, Jaswal N. The Health-Seeking Behavior of Leprosy Patients: An explanatory model. hcs. 2013;4(1):52–65. doi: 10.5195/hcs.2013.105

[6] Chala TK, Gudina EK, Adorjan K, Froeschl G. Exploring the health-seeking journeys of individuals affected by leprosy: Lived experiences in selected urban rehabilitation centers in Ethiopia. PLoS Negl Trop Dis. 2026;20(2):e0013938. doi: 10.1371/journal.pntd.0013938 41701794 PMC12931759

[7] Ilozumba O, Lilford R. Self-care programmes for people living with leprosy: a scoping review. Lepr Rev. 2021;92(4):317–37.

[8] Stuetzle SCW, Bonkass A-K, van Brakel WH, Schoenmakers A, Fastenau A. Self-healing in leprosy: A systematic review. PLoS Negl Trop Dis. 2024;18(9):e0012434. doi: 10.1371/journal.pntd.0012434 39264863 PMC11392240

[9] Willis M, Asboeck A, Amorim BBC, Deps PD, Fastenau A, Boeckmann M. Self-care interventions to reduce, prevent or monitor physical disability in those affected by leprosy: Protocol for a systematic review. PLoS One. 2025;20(10):e0330477. doi: 10.1371/journal.pone.0330477 41166248 PMC12574909

[10] Amenu A, Nash J, Tamiru T, Byass P. Patterns of health-seeking behaviour amongst leprosy patients in former Shoa Province, Ethiopia. Ethiopian Journal of Health Development. 2000;14(1). doi: 10.4314/ejhd.v14i1.9929

[11] Urgesa K, de Bruijne ND, Bobosha K, Seyoum B, Mihret A, Geda B, et al. Prolonged delays in leprosy case detection in a leprosy hot spot setting in Eastern Ethiopia. PLoS Negl Trop Dis. 2022;16(9):e0010695. doi: 10.1371/journal.pntd.0010695 36094952 PMC9499267

[12] Susanti IA, Mahardita NGP, Alfianto R, Sujana IMIWC, Siswoyo, Susanto T. Social stigma, adherence to medication and motivation for healing: A cross-sectional study of leprosy patients at Jember Public Health Center, Indonesia. J Taibah Univ Med Sci. 2017;13(1):97–102. doi: 10.1016/j.jtumed.2017.06.006 31435310 PMC6695038

[13] Santos GMCD, Byrne RL, Cubas-Atienzar AI, Santos VS. Factors associated with delayed diagnosis of leprosy in an endemic area in Northeastern Brazil: a cross-sectional study. Cad Saude Publica. 2024;40(1):e00113123. doi: 10.1590/0102-311XEN113123 38198383 PMC10775965

[14] Dijkstra JIR, Brakel WH, Elteren M. Gender and leprosy-related stigma in endemic areas: A systematic review. Lepr Rev. 2017;88(3):419–40.

[15] Schoenmakers A, Hambridge T, van Wijk R, Kasang C, Richardus JH, Bobosha K, et al. PEP4LEP study protocol: integrated skin screening and SDR-PEP administration for leprosy prevention: comparing the effectiveness and feasibility of a community-based intervention to a health centre-based intervention in Ethiopia, Mozambique and Tanzania. BMJ Open. 2021;11(8):e046125. doi: 10.1136/bmjopen-2020-046125 34446483 PMC8395349

[16] Wilder-Smith AB, Li J, Fuller LC, Anwar SN, van Rooijen E, Freeman EE, et al. Strengthening dermatology capacity and competency in resource-limited settings: A viewpoint. PLoS Negl Trop Dis. 2025;19(11):e0013720. doi: 10.1371/journal.pntd.0013720 41270034 PMC12637970

[17] WHO Team. Towards zero leprosy global leprosy (Hansen’s disease) strategy 2021-2030. 8. World Health Organization. 2021.

