# Supplementary material for: Barriers to biomedical care: Qualitative insights into health-seeking behaviors and systemic challenges among people affected by leprosy in Tanzanian communities
Source: PLoS Negl Trop Dis. 2026 Oct 15;20(10):e0014806. doi: 10.1371/journal.pntd.0014806 (PMC13693982; doi:10.1371/journal.pntd.0014806)
Supplement: Supplementary file 1 [file pntd.0014806.s001.docx]

**FACTORS AND IMPACTS ASSOCIATED WITH GENDER-BASED VIOLENCE AMONG WOMEN AFFECTED BY LEPROSY IN TANZANIA**

***IDI – GBV PARTICIPANTS TOOL GUIDE***

**Date: ………	IDI No: ………… Facilitator Initials: ………… Location/Venue: ………..**

**Marital status: ………… Occupation: …………Age: …………. Sex ………..**

**General knowledge of Leprosy**

1. Have you ever heard about Leprosy?
2. Do you know what causes Leprosy? If yes, mention…
3. How is Leprosy transmitted?
4. Who is at risk of Leprosy (Probe elders, young people, anyone, others (specify))
5. How do you know if someone has Leprosy?
6. Based on your experience, is there any cure for leprosy?
7. Is Leprosy preventable? (Probe: wearing mask, isolation, others (specify)
8. What do people in your community say about Leprosy?

**Knowledge and perception of Gender-Based Violence among women affected by leprosy**

9. What do you understand about GBV?
10. Do women affected by leprosy experience GBV? If yes, **probe:** why?
11. What kind/form of GBV do people experience just because they are affected by leprosy?

***Probe:***

•	Sexual violence – (avoid sexual intercourse, extra marital, cheating)

•	Physical violence – (beating, hitting, punching, kicking, pushing)

•	Emotional violence – (given nick names, bullying, insults related to this ability like skin condition, etc, isolating them from family, friends)

•	Economical violence – (restrictions to financial resources)

a)	The first time you experienced GBV, who was the person responsible, and How old were you when it happened?

12. What do you think are the reasons associated with GBV among people affected with leprosy? ***Probe*** if the reasons are: Poor knowledge about leprosy, Alcohol use, GBV being considered a private matter, Cultural permissiveness-men physical aggression against women, Age (young age).
13. How did you handle the situation against GBV? Probe: (keeping quiet, fighting, divorce)
14. Can you tell me which of the types of GBV mentioned are the most common types within your community?

**Practices on seeking for care**

15. Where do you often seek help against GBV? **Probe:** (family member, friends, police station, village leaders, religion leaders)
16. On average, what is the time for someone who has experienced GBV to decide to report and seek help? (very early or too late)
17. For those who delay in seeking help, what are the factors/reasons behind the delay? **Probe:** (family provider, fear about divorce, believing they will change, love.)
18. Does GBV have any effect on you? If yes, Probe the effects
19. What are the effects of GBV on health-seeking behavior among people affected by leprosy? Probe: If (Insult and isolation can be a barrier to timely health-seeking) or Power dynamics (required partner's permission to disclose the disease)
20. What do you think should be done to prevent GBV from women affected by leprosy?

Any opinions /suggestion/comments

**THANK YOU**
