# Supplementary material for: Barriers to biomedical care: Qualitative insights into health-seeking behaviors and systemic challenges among people affected by leprosy in Tanzanian communities
Source: PLoS Negl Trop Dis. 2026 Oct 15;20(10):e0014806. doi: 10.1371/journal.pntd.0014806 (PMC13693982; doi:10.1371/journal.pntd.0014806)
Supplement: Supplementary file 2 [file pntd.0014806.s002.docx]

**FACTORS AND IMPACTS ASSOCIATED WITH GENDER-BASED VIOLENCE AMONG WOMEN AFFECTED BY LEPROSY IN TANZANIA**

***IDI TO DTLC AND FGD TO CHW - TOOL GUIDE***

**Date: ………	FGD No: ………… Facilitator Initials:………… Location/Venue:………..**

**Marital status: ………… Occupation: …………Age: …………. Sex ………..**

**General knowledge of Leprosy**

1. Have you ever heard about Leprosy?
2. Do you know what causes Leprosy? If (If not mentioned, probe witchcraft/demons, curse, inheritance, infection)
3. Do you think epilepsy can be transmitted from one person to another? (If yes, probe how and why so for each mentioned)
4. Who is at risk of Leprosy (Probe elders, young people, anyone, others (specify))
5. How do you know if someone has Leprosy?
6. Who normally diagnoses or confirms if someone has Leprosy? (Probe: family members, traditional healers, health facility staff, etc.)
7. Based on your experience, is there any cure for leprosy?
8. Is Leprosy preventable? (Probe: wearing mask, isolation, others (specify)
9. What do people in your community say about Leprosy? (Probe: the local terminologies, jokes, insulting language, etc)

***Treatment perception (asked only to DTLC NOT CHWs and community members)***

10. *Normally, how are persons affected with leprosy seen in your workplace compared to patients with other diseases? (Probe: disabled, useless, weak, burden, etc.)*
11. *average, what time is taken from the time of suspecting epilepsy until someone reports to a health facility? (Probe: very early, too late, etc.) Why?*
a) *If there are delays, ask what are the reasons behind late reporting at the health facility?*
b) *What are the implications for (very early, too late) reporting at the health facility?*
c) *In most cases, who takes the patient to the health facility?*

*12.Is there a cure, from your experience? (Probe: short-term or long-term, medications, etc.)*

*a)	If yes, is it accessible, affordable, and available to patients*

*13. What are the barriers encountered when managing people affected with leprosy in the Health facility services (Probe: Professional staff, equipment, medications, continual education, regulations)*

*14. Do you know people affected with leprosy experienced GBV? If yes please explain*

- *After they reported GBV to you what action do you take?*

**Perceived Gender-Based Violence among people affected by leprosy**

12. What do you understand about GBV?
13. Do the people affected by leprosy experience GBV? If Yes, pro**be:** why?
14. What kind/form of GBV do people affected by leprosy experience? **Probe:**

- Sexual violence – avoid sexual intercourse, extramarital
- Physical violence – beating, hitting, punching, kicking, throwing/pushing
- Emotional violence – given nicknames, insulted related to disability like skin condition, etc
- Economical violence –restrictions to financial resources

**Factors associated with the occurrence of GBV among people affected with leprosy**

15. What do you think are the reasons associated with GBV among people affected with leprosy? **Probe** if the reasons are**:** Poor knowledge about leprosy, Alcohol use, GBV be considered a private matter, Cultural permissiveness-men physical aggression against women, Age (young age).

**Impacts of GVB on health seeking among people affected by leprosy**

16. Does GBV have any effect on people with leprosy?
17. What are the effects of GBV on health-seeking behavior among people affected by leprosy? **Probe:** If

- Insult, verbal violence, and isolation can be a barrier to timely health-seeking
- Power dynamics (required partner's permission to disclose about the disease)

**Impacts on well–being** (health, comfortable, happy)

18. What are the effects of GBV on the well–being of people affected by leprosy? **Probe:**

- Psychological impact due to insults related to their disease
- Collapse of family structure
- Unable to take part in community engagement
- Loss of financial stability

19. What should be done to prevent GBV among people affected by leprosy?
20. Any opinion/suggestion/comment

**THANK YOU**
